# Quality of Life Assessment in Diabetic Patients Using a Validated Tool in a Patient Population Visiting a Tertiary Care Center in Bhubaneswar, Odisha, India

**DOI:** 10.1155/2020/7571838

**Published:** 2020-12-29

**Authors:** Dayanidhi Meher, Sonali Kar, Mona Pathak, Snigdha Singh

**Affiliations:** ^1^Department of Endocrinology, Kalinga Institute of Medical Sciences (KIMS), Bhubaneswar 751024, India; ^2^Department of Community Medicine, Kalinga Institute of Medical Sciences (KIMS), Bhubaneswar 751024, India; ^3^Department of Biostatistics, Kalinga Institute of Medical Sciences (KIMS), Bhubaneswar 751024, India

## Abstract

Odisha has 4.2 million diabetic patients against the country's 70 million with an urban prevalence of nearly 15.4%. Diabetes is affecting younger age groups, thus having a crucial impact on quality of life of the affected. A qualitative endeavour was attempted at the diabetic clinic of a tertiary care set up in the capital city of Bhubaneswar to create a diabetic surveillance data assembly, wherein subjects above 18 years of age and newly diagnosed or on follow-up, after obtaining informed consent, were made to respond to a quality of life (QOLID) validated tool. The pretested tool has 8-domain role limitation due to physical health, physical endurance, general health, treatment satisfaction, symptom botherness, financial worries, emotional/mental health, and diet advice tolerance. The validated tool had 34 items (questions) that were selected to represent these domains on the basis of extraction communality, factor loading, and interitem and item-total correlations. The final questionnaire had an overall Cronbach's alpha value of 0.894 (subscale: 0.55 to 0.85), showing high internal consistency in the current study population. A score for each domain was calculated by simple addition of items scores. Each individual domain score was then standardized by dividing by maximum possible domain score and multiplying by 100. All individual standardized domain scores were then added and divided by 8 (number of domain) to obtain an overall score. The data collection was done for 400 patients as an interim analysis. Univariate and subsequently multivariate analysis was performed to decide the predictors that affected quality of life. Age over 50 years (OR = 1.81, CI 1.12–2.93; *p*=0.014), female gender (OR = 2.05, CI 1.26–3.35; *p*=0.004), having foot complications (OR = 2.81, CI 1.73–4.55; *p* < 0.001), and having depression (OR = 1.88, CI 1.15–3.06, *p*=0.011) emerged as predictors of poor QOLID scores. The tool can be made a subtle part of chronic case management of diabetes to ensure patient's participation in the treatment of the disease and to create a database that can redefine diabetic care in India to suit the diverse regional settings in the country.

## 1. Introduction

Diabetes and its management have become a challenge in the current scenario, where in India is being predicted as the diabetic capital, and by 2025, we are expected to have a staggering 70 million diabetics in the country [1, 2]. In the context of the epidemiological pattern of the disease in Asians, they are seen to have a proven susceptibility to early onset of disease, higher insulin resistance, existence of comorbidities, and perhaps lack of holistic healthcare systems [3, 4]. Hence in the recent times, research has picked up in these populations and the management is also being directed in a way to address the disease holistically. Long-term dietary restrictions, follow-ups, and adherence to medications are most likely to affect the lifestyle of the patient. Noncommunicable diseases like diabetes have made it a popular and pertinent necessity to assess quality of life of the individual, after the disease onset/diagnosis to optimize or maximize the management outputs. Quality of life (QOL) has been defined by WHO as “individuals' perceptions of their position in life in the context of the culture and value systems in which they live in relation to their goals, expectations, standards, and concerns” [5]. The essence of this definition also elucidates the subjectivity and complexity of developing measures of QOL, which are primarily questionnaire-based tools that are expected to include generic and disease specificity as well as the social milieu of the patient. Thus, a host of QOL quality of life questionnaires including WHOBREF [5], DQLCTQ (Diabetes Quality of Life Clinical Trial Questionnaire) [6], and DQOL (Diabetes Quality of Life) [7] have attempted to link the QOL assessment to disease management in an effort to identify and enhance patient's health-related quality of life issues, which in turn have the potential to improve compliance and thus their metabolic status. Most of these questionnaires have been validated in the Western population and then have been utilized in the Indian population in select study settings. In a unique effort, all 3 questionnaires were assessed and a cumulative complete QOL questionnaire (QOLID) was validated by Sitaram Bhartia Institute of Science and Research [8] in a scientifically discrete two-phase pattern to finalize a questionnaire with 34 items covering eight domains. This attempted to comprehensively cover aspects of quality of life, namely, role limitations due physical health, physical endurance, general health, treatment satisfaction, symptom frequency, financial worries, mental health, and diet advice satisfaction. The scale was more true to the Indian scenario in terms of inclusion of financial worries and mental health which were subtly included in other scales, being more of a Western origin. The current study was taken up in a state-of-art diabetic clinic of a tertiary care centre in East India, that is, Bhubaneswar, Odisha, a state in India which along with West Bengal is hyped as the diabetic bowl of the country.

### 1.1. Aims and Objectives

1. To assess the diabetic patients (ambulatory) coming to the tertiary care center for quality of life (QOLID) as per the 8-domain questionnaire tool validated for Indian population
2. To find out the determinants and risk factors attributing to the QOL scores

## 2. Methodology

The study was undertaken as a research component aimed to qualitatively improve upon the management of the diabetics, attending the state-of-art center of Kalinga Institute of Medical Sciences, Bhubaneswar, as a collaborative approach between the Endocrinology and Department of Community Medicine. After due ethical clearance since 2016, the study data include the patient pool from December 2019 to May 2020 (6 months) in the current article.

The study tool was the questionnaire validated by Sitaram Bhartia [8] which was used for the QOL assessment and tested for its face and construct validity by the study team comprising of public health, a biostatistician, and an endocrinologist. The tool had 34 items (questions) that were selected to represent these domains on the basis of extraction communality, factor loading, interitem, and item-total correlations. The final questionnaire had an overall Cronbach's alpha value of 0.894 (subscale: 0.55 to 0.85), showing high internal consistency, thus hinting at appropriateness for use in the given population.

To decide and considering the recommendation of 10 samples per item for validation of questionnaire [9–11] for 34 items in our scale, we need a minimum of completed response for 340 subjects. Within the stipulated time period of the study, i.e., 6 months, we could draw complete data for 400 subjects.

The inclusion criteria for participation in the study were fixed as a diagnosed diabetic (any type I or II), attending the art-of-care diabetic clinic, above 18 years of age, who gives informed consent for the study, who is not suffering from debilitating or life-threatening complications of the disease, and who does not warrant admission to the hospital and answers all questions completely. Incompletely answered QOL questionnaires, 28 of them, were deleted from the study analysis. The exclusion criteria were nonwillingness to participate in the study and having end-stage diabetic disease. Here, the discretion of the consulting endocrinologist also contributory to decide for inclusion in the study.

A team of data collectors were trained in the QOL questionnaire, which also had sections on sociodemographic data such as age, sex, urban/rural residence, education, and years since definitive diagnosis of diabetes, treatment modality, and adherence to management and a Diabetic Care Scale.

In diabetes, like in any chronic NCD, a lot of emphasis is laid on compliance to treatment advice which in our case is adapted from the 4-item Morisky Medication Adherence Scale (MMAS) which has been widely used in India to assess the same in cardiovascular patients [12, 13]. As the MMAS scale is paid and not for openusage, we modified the questions as if the subject complied to all advice given by the treating physician in terms of medications (oral/insulin/both) and diet, since diagnosis and pescription for each single day. If the answer was yes, it was taken as complete compliance and any other answer was taken as noncompliant.

The abovementioned questions were answered yes/no. Every yes adds up to one score. Hence, the total score is 4. The top score is 0 which means high adherence, 1 to 2 suggests medium adherence and 3 to 4 low adherence [13]. This was taken as a risk variable affecting QOL.

Physical attributes such as Basal Metabolic Rate (BMI), systolic blood pressure recording (taken by a digital BP machine as a measure of average of 3 readings in the left upper arm when the subject is at rest), Fasting Blood Sugar Values (FBS in most current lab reports available with the study subject) were also considered as risk factors affecting the QOL. BMI has been classified as per WHO [14] and the cutoff for FBS was taken as 100 mg/dl [15].

A Diabetic Care Scale [16], with total score 60 and median in this population, i.e. 35, was taken for categorizing good care (35 and above) and bad care. It was taken as a proxy measure of the patient's perception of the quality care provided by the health provider. Being a chronic disease, it is very essential to build a measure of patient's perception of the healthcare provider and the care being offered to him/her. This measure was incorporated as a risk factor to help improve the patient-physician/nurse interactions.

The QOLID score for each of the eight domains was calculated by simple addition of items scores. Each individual domain score was then standardized by dividing by maximum possible domain score and multiplying by 100. All individual standardized domain scores were then added and divided by 8 (number of domain) to obtain an overall score [8]. As all the domain scores are reported as continuous scores with SD and follow normal distribution, T-test was used to identify association between aggregate QOL domain scores and the sociodemographic or other individual variables. Step-wise logistic regression analysis with forward selection and backward elimination with removal as well as inclusion probability of 5% was used to identify the independent predictors of quality of life. All the variables which were significant under univariate analysis were considered as candidate variable for multivariable analysis. All the results were interpreted at 5% level of significance and Stata 15.1, StataCorp, Texas, was used for the analysis.

## 3. Results

400 diagnosed cases of diabetes seeking treatment in the state-of-art center of a tertiary care center were assessed for quality of life (QOL) using the QOLID tool of 8 domains with 34 items which is validated in the Indian population, as per the study design.

Table 1 shows the domain-wise scores as well as the overall QOLID scores for the given sample of 400 diabetics, with the mean score being 60.56 ± 10.05. The scores were segregated for gender and age, and it was noted that, for males the overall QOLID scores were 62.238 ± 9.032 vs. 57.821 ± 11.023, suggesting better quality of life in males and same was seen in the age break-up wherein less than 50 years scores were 62.084 ± 9.67 vs. 59.134 ± 10.251, as noted for those aged more than 50 years.

If domain-wise scores were to be considered, high scores were noted for the domain role limitation due to physical health which was 79.36 ± 18.03 and was noted far better in males vs. females (82.607 ± 15.197 vs. 74.057 ± 20.864) and similar advantage was observed in less than 50 years to the ones above 50 years (82.038 ± 16.749 vs. 76.82 ± 18.881). Males also score better in terms of emotional and mental health than the female counterparts. Least scores were observed for domain of treatment satisfaction which were 35.97 ± 8.89, wherein females and over 50 years were seen to be marginally scoring better than the counterparts. This was also the only domain wherein the females' mean score was one point higher than the males; in all other domains, the means were less than males, as can be seen in Table 1. This gives subtle hints to the managing team on the domains to focus for counselling and sensitive care.

Table 2 presents the univariate analysis and subsequent multivariate analysis done by stepwise logistic regression analysis with forward selection as well as backward elimination with removal of factors coming significant in univariate analysis. For both analysis and inclusion, a probability of 5% was used to identify the independent predictors of quality of life for the respondents. The QOLID scores were converted into categorical variable by obtaining the mean score and dividing the group into those who got a score above the mean and those below the mean for the regression analysis. Age over 50 years (OR = 1.81, CI 1.12–2.93; *p*=0.014), female gender (OR = 2.05, CI 1.26–3.35; *p*=0.004), having foot complications (OR = 2.81, CI 1.73–4.55; *p* < 0.001), and having depression (OR = 1.88, CI 1.15–3.06, *p*=0.011) emerged as predictors of poor QOLID scores. Interestingly, the Diabetic Care Scale which was taken as a proxy measure of patient's satisfaction of the care provider emerged as the strongest predictor of QOL scores, with 6.5 times more possibility of bad QOL score in case of dissatisfaction with the provider's care.

## 4. Discussion

The current study is an effort to qualitatively improvise the chronic management of diabetes in a state-of-art center. Odisha, a state in East India with its capital Bhubaneswar, is now emerging as the hub for health and education. The capital houses three medical colleges, besides corporate hospitals which add promise to offer comparable healthcare to the clients. The improved access to lab facilities and knowledge on the disease has prompted people to visit these state-of-art centers offering diabetic care. Being a chronic disease, which stays for a lifetime and warrants care throughout, it affects the quality of life drastically as proven in several studies in terms of treatment patterns and glycemic controls [18–20].

As discussed before, the QOLID questionnaire offers a holistic insight into patient's needs and expectations from the disease management in terms of financial, persisting or deteriorating symptoms, and role limitation and not just physical and psychological aspect of life as offered by WHO BREF HRQOL (health-related QOL). As discussed in results, we saw that the overall 8-domain QOLID score was 60.56 ± 10.05, which is comparable to mean total score of 58.05 (95% CI, 22.18–93.88) reported in a study conducted in rural population of South India and another study from Thiruvananthapuram, which showed that 62% of the diabetics reported good QOL [21, 22]. However, instruments used in these studies were the WHO BREF questionnaires. The important result reflected is the poor scores in treatment satisfaction domain in this study, overall being 35.97 ± 8.89, while in a South Indian study [21], physical QOL had one of the lowest scores of 58.64 + 18.54. This difference could be for the regional variations in disease management. Odisha has predominantly rural population and hence adherence and access to treatment facilities are poor.

A pertinent finding in the study is the predictor of foot complications for poor QOLID scores (2.81; CI 1.73–4.55) which has again established the need for foot care education among the East India population [23]. The poor mental health also attributed to low scores in this study, while in the South Indian study, 69% had high psychological scores. This can be explained by the rural background of the study population in the latter study, wherein the social support system and family structure are very strong. The Bhubaneswar study population were more urban and semiurban who could afford a visit to the tertiary healthcare.

The higher age (above 50 years) and female gender were also vulnerable to poor QOLID scores, which is a universal finding in all studies conducted on QOL in India [21, 22, 24, 25].

In this study, the model tries to find out the risk of noncompliance with QOLID, but the results came insignificant for poor scorers with poor or noncompliance. This could be because the disease permits the individual to live a normal life, until the onset of severe complications. However, from management point of view, it is important to know the subject's compliance to prescription and the reasons thereof.

Thus, the study brings out strongly that diabetes is a multifactorial disease and its management is not limited to treatment in terms of assigning medications and the social milieu. The strength of the study is the range of variables adjusted in the step-wise univariate and multivariate analysis to arrive at predictors. As a self-audit, the Diabetic Care Scale was also taken up as a risk factor which has not been assessed in any study and this emerged as the strongest predictor in this population, suggesting that the patient's perception of the healthcare provider and the care being offered has most bearing on the QOL scores.

The study has limitations in terms of using the same questionnaire for type 1 and type 2 diabetes, while most studies done segregated analysis for this too. Another one is the inability to follow-up the subjects in the subsequent visits to check for variations in the QOLID scores. The study is ongoing and is offering key hints to improve the range of care and management being offered at only one tertiary care center in Bhubaneswar, Odisha, which too is a major limiting factor. This exercise can be made an inbuilt part of diabetic care to offer more personalized and satisfactory care to the diabetic patients.

## Figures and Tables

**Table 1:** QOLIID scores: total score and domainwise score presented agewise and genderwise.

Domains of QOLID	Sample (*N* = 400) (mean ± SD)	Gender (*N* = 400)		Age (*N* = 399)	
Score	Aggregate	Male	Female	Less than 50	More than 50
Treatment satisfaction	35.97 ± 8.89	**35.60** **±** **8.29**	**36.572** **±** **9.777**	**35.593** **±** **8.625**	**36.349** **±** **9.145**
General health	53.97 ± 16.8	55.215 ± 16.008	51.929 ± 17.88	55.578 ± 17.234	52.394 ± 16.282
Symptom botherness	74.72 ± 22.81	75.510 ± 22.456	73.421 ± 23.385	75.27 ± 23.092	74.368 ± 22.509
Financial worries	57.91 ± 21.11	59.536 ± 20.912	55.263 ± 21.219	57.227 ± 21.132	58.495 ± 21.147
*Emotional and mental health*	79.35 ± 14.11	**81.032** **±** **12.493**	**76.605** **±** **16.077**	**79.709** **±** **14.303**	**79.048** **±** **13.977**
Diet satisfaction	69 ± 12.58	69.569 ± 12.984	68.070 ± 11.877	69.568 ± 12.504	68.446 ± 12.686
Physical endurance	70.18 ± 23.78	74.435 ± 21.522	63.223 ± 25.649	77.288 ± 22.031	63.495 ± 23.523
*Role limitation due to physical health*	79.36 ± 18.03	**82.607** **±** **15.197**	**74.057** **±** **20.864**	**82.038** **±** **16.749**	**76.82** **±** **18.881**
Total score quality of life	60.56 ± 10.05	62.238 ± 9.032	57.821 ± 11.023	62.084 ± 9.67	59.134 ± 10.251

Bold values indicate QOLID scores that show major difference and explained in Section 3.

**Table 2:** Associating factors affecting QOLID scores in the sample and multivariate analysis determining the final predictors.

Factors	Number (%)	Univariate		Multivariate	
		Odds ratio (95% CI)	*p* value	Odds ratio (95% CI)	*p* value
Sociodemographic characteristics					
*Gender*					
Male	248 (62.00)	1.00	—	1.00	—
Female	152 (38.00)	1.96 (1.30–2.96)	0.001	2.05 (1.26–3.35)	0.004
*Age*					
Less than 50	193 (48.37)	1.00	—	1.00	—
More than 50	206 (51.63)	1.64 (1.06–2.52)	0.026	1.81 (1.12–2.93)	0.014
*Education*					
Illiterate	18 (4.50)	1.00	—	—	—
Primary school	12 (3.00)	0.77 (0.16–3.74)	0.745	—	—
Middle school	35 (8.75)	0.41 (0.12–1.39)	0.151	—	—
Secondary school	158 (39.50)	0.43 (0.15–1.28)	0.132	—	—
Higher secondary and above	177 (44.25)	0.28 (0.10–0.83)	0.021	—	—
*Residence*					
Urban	—	1.00	—	—	—
Rural	—	1.10 (0.74–1.63)	0.630	—	—
Individual subject characteristics					
*Body mass index*					
Normal	134 (33.50)	1.00	—	—	—
Overweight	178 (44.50)	0.55 (0.35–0.87)	0.010	—	—
Obese	88 (22.00)	0.77 (0.45–1.33)	0.354	—	—
*Waist circumference* (cm) (Ref. [17])					
Normal (M 102 cm; F 88 cm)	48 (12.00)	1.00	—	—	—
Abnormal (M > 102 cm; F > 88 cm)	352 (88.00)	0.98 (0.53–1.79)	0.941	—	—
*Current tobacco chewing*					
Yes	108 (27.00)	1.00	—	—	—
No	263 (65.75)	1.30 (0.83–2.04)	0.255	—	—
Sometimes	29 (7.25)	1.54 (0.67–3.51)	0.306	—	—
*Current use of smoking tobacco*					
Yes	24 (6.00)	1.00	—	—	—
No	359 (89.75)	1.69 (0.72–3.97)	0.225	—	—
Sometimes	17 (4.25)	1.48 (0.42–5.23)	0.541	—	—
*Current use of alcohol*					
Yes	18 (4.50)	1.28 (0.37–4.44)	0.700	—	—
No	350 (87.50)	2.77 (1.24–6.15)	0.012	—	—
Sometimes	17 (4.25)	1.00	—	—	—
*Duration of diabetes*					
Less than 7 years	202 (50.50)	1.00	—	—	—
More than 7 years	198 (49.50)	1.55 (1.04–2.30)	0.028	—	—
*Fasting blood sugar*					
<130	190 (47.86)	1.00	—	—	—
≥130	207 (52.14)	1.37 (0.92–2.03)	0.117	—	—
*Compliance to management*					
0 (complete compliance)	255 (63.75)	1.00	**0.133**	—	—
1–4 (noncompliant)	145 (36.25)	1.37 (0.91–2.06)	—	—	—
*Treatment*					
Diet only	13 (3.25)	—	—	—	—
	255 (63.75)	1.00	—	—	—
Medicine with diet	—	2.69 (0.72–10.02)	0.139	—	—
Insulin with diet	49 (12.25)	6.27 (1.52–25.90)	**0.011**	—	—
Medicine and insulin with diet	83 (20.75)	4.80 (1.23–18.76)	**0.024**	—	—
*Complications of eye*					
No	165 (41.25)	1.00	—	—	—
Yes	235 (58.75)	2.38 (1.58–3.59)	<0.001	—	—
*Complications of ear*					
No	327 (81.75)	1.00	—	—	—
Yes	73 (18.25)	2.26 (1.33–3.8)	0.002	—	—
*Complications of kidney*					
No	39.75 (90.75)	1.00	—	—	—
Yes	37 (9.25)	2.62 (1.26–5.47)	0.010	—	—
*Complications of neuro*					
No	111 (27.75)	1.00	—	—	—
Yes	289 (72.25)	1.95 (1.25–3.06)	0.003	—	—
*Complications of foot*					
No	241 (60.25)	1.00	—	1.00	—
Yes	159 (39.75)	3.09 (2.03–4.70)	<0.001	2.81 (1.73–4.55)	<0.001
*Complications of depression*					
No	164 (41.00)	1.00	—	1.00	—
Yes	236 (59.00)	2.91 (1.92–4.41)	<0.001	1.88 (1.15–3.06)	0.011
*Recovery*					
No hospitalization	265 (66.25)	1.00	—	—	—
Completely	77 (19.25)	1.46 (0.88–2.43)	0.143	—	—
Partially	46 (11.50)	2.40 (1.25–4.63)	0.008	—	—
No relief at all	12 (3.00)	14.13		—	—
		(1.80–111.01)	0.012	—	—
*Morbidity of HTN*					
No	178 (44.50)	1.00	—	—	—
Yes	222 (55.50)	1.45 (0.97–2.15)	0.067	—	—
*Morbidity of others*					
No	163 (40.75)	1.00	—	—	—
Yes	237 (59.25)	0.71 (0.47–1.05)	0.091	—	—
*Diabetic Care Scale*					
Good (>35)	208 (52.13)	1.00	—	1.00	—
Bad (<35)	191 (47.87)	6.75 (4.35–10.46)	<0.001	**6.50 (4.01-10. 52)**	**<0.001**

Bold values indicate the the significant *p* values (*p*< or=0.05 at 95% CI) for factors after univariate and multivariate analysis. Details are provided in Section 3.

## Data Availability

The excel file (Supplementary Materials) bearing the collated data of all subjects along with the questionnaire is shared for the journal's kind reference.

## Supplementary Materials

Supplementary Materials The collated data of all subjects along with the questionnaire used for this study.
