# Supplementary material for: Quality of Life Assessment in Diabetic Patients Using a Validated Tool in a Patient Population Visiting a Tertiary Care Center in Bhubaneswar, Odisha, India
Source: ScientificWorldJournal. 2020 Dec 29;2020:7571838. doi: 10.1155/2020/7571838 (PMC7785381; doi:10.1155/2020/7571838)
Supplement: Supplementary Materials — The collated data of all subjects along with the questionnaire used for this study. [file 7571838.f1.docx]

**Diabetes surveillance**

**KIMS Diabetic Clinc(Dr.Meher/Dr. Sonali)**

**SERIAL NO. :**

1. **Patient information:**

| **SL. No.** | **Variables** | **Responses** | | | | |
| --- | --- | --- | --- | --- | --- | --- |
| **1.** | Name |  | | | | |
| **2.** | Gender | 1. Male | | 1. Female | | |
| 3. | Age (years) | 1. Less than 30 2. 30-50 3. 51-70 4. More than 70 | | | | |
| **4.** | Residence | 1. Urban | | | 1. Rural | |
| **5.** | Permanent Residence ( District) |  | | | | |
| **6.** | Occupation |  | | | | |
| **7.** | Education | 1. Illiterate 2. Primary school 3. Middle school 4. Secondary school 5. Higher secondary and above | | | | |
| 8. | Tobacco   1. Chewing   2. Smoking | 1. Yes   1.Yes | 1. No   2.No | | | 1. Sometimes   3.Sometimes |
| 9. | Alcohol intake | 1.Yes | 2.No | | | 3.Sometimes |

1. **History and current status of diabetes:**

|  | **Parameters** | **Responses** | | |
| --- | --- | --- | --- | --- |
| 1. | Diagnosis (complete) |  | | |
| 2. | First diagnosed on / (Duration of diabetes) |  | | |
| 3. | Facility where 1^st^ diagnosed |  | | |
| 4. | Treatment | 1. Diet modification only | 1. Oral Medicines | 1. Insulin |
| 4.1 | If medicines: Name the medicine(put generic medicines only) |  | | |

**Medicine adherence :**

**Medication Compliance Scale**

1.Forgot to take medicines ever- yes / no

2.Careless about taking medications- yes / no

3.When feeling better stopped taking medicines and restarted after counseling- yes / no

4.When feeling worse, stopped taking medicines and restarted after counseling- yes / no

1. **General and lab investigation information:**

| **Sl.No** | **Parameters** | **Values/Response** | | | |
| --- | --- | --- | --- | --- | --- |
| 1. | Height |  | | | |
| 2. | Last FBS |  | | | |
|  |  | **Current** | **1^st^ follow up** | | **2^nd^ follow up** |
| 1. | Weight |  |  | |  |
| 2. | BMI |  |  | |  |
| 3. | Waist circumference |  |  | |  |
| 4. | Blood Pressure |  |  | |  |
| 5. | FBS |  |  | |  |
| 6. | Cholesterol |  |  | |  |
| 7. | HbA1C |  |  | |  |
| 8. | Lipid profile  1.HDL  2.LDL  3.TG |  |  | |  |
| 9. | Serum urea |  |  | |  |
| 10. | Serum creatinine |  |  | |  |
| 11. | UACR |  |  | |  |
| 12. | Any other co-morbidities: | 1. HTN 2. PCOD 3. Mental health 4. Cancer 5. Any other ………. | 1. HTN 2. PCOD 3. Mental health 4. Cancer 5. Any 6. other ………. | | 1. HTN 2. PCOD 3. Mental health 4. Cancer 5. Any 6. other ………. |
| 13. | Any hospitalizations in last 2 years | 1. Yes | 2. No | | |
| 13.1 | If yes, state the reason |  | | | |
| 13.2 | State if recovered | 1. Completely 2. Partially 3. No relief at all | | | |
| 14. | Any Complications | 1. Eye 2. Foot 3. Kidney 4. Ear 5. Neuropathy | | 1. Depression | |

**DIABETES CARE SCALE**


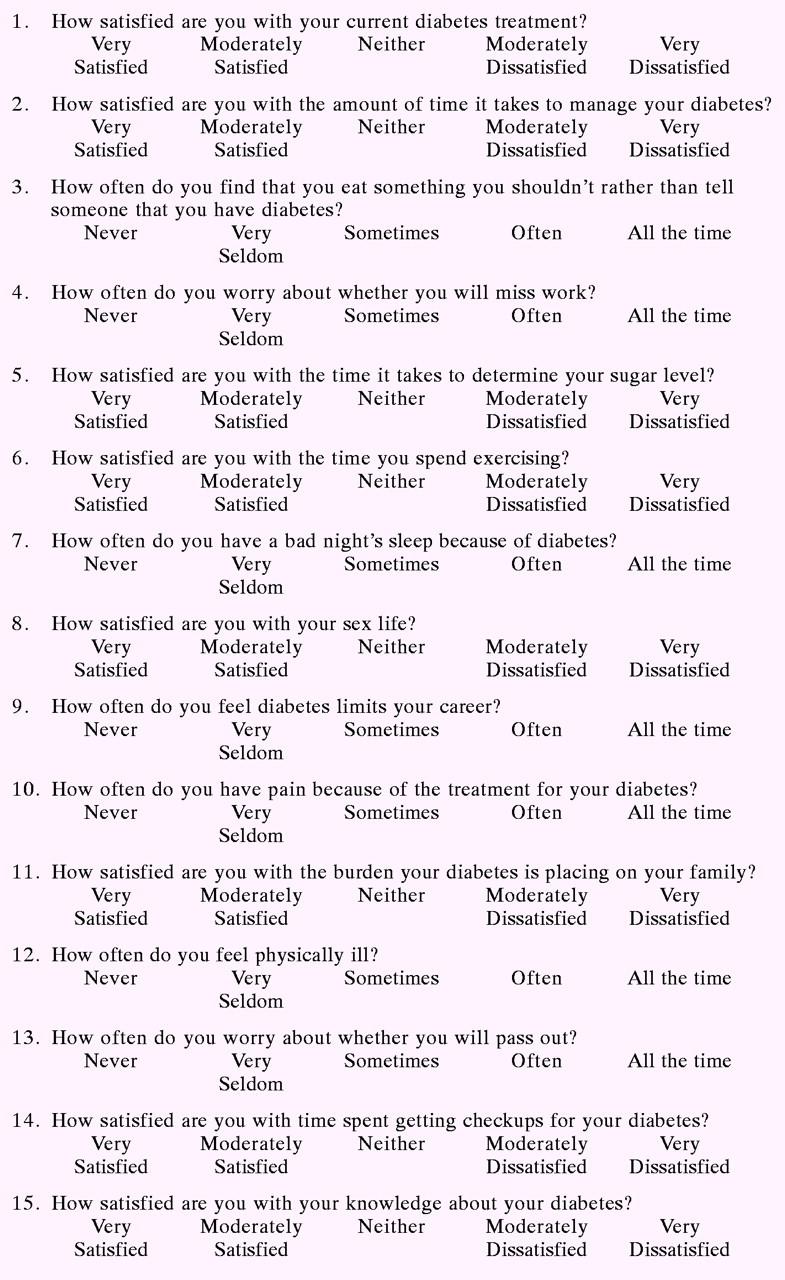


**QOLID for assessment of Quality of Life in Diabetes**

**Treatment Satisfaction**

1. How satisfied are you with your current diabetes treatment?

| Very dissatisfied | Moderately dissatisfied | Neither satisfied nor dissatisfied | Very Good | Excellent |
| --- | --- | --- | --- | --- |
| 1 | 2 | 3 | 4 | 5 |

2. How satisfied are you with amount of time it takes to manage your diabetes?

| Very dissatisfied | Moderately dissatisfied | Neither satisfied nor dissatisfied | Very Good | Excellent |
| --- | --- | --- | --- | --- |
| 1 | 2 | 3 | 4 | 5 |

3. How satisfied are you with the amount of time you spend getting regular checkups (once in 3 months)?

| Very dissatisfied | Moderately dissatisfied | Neither satisfied nor dissatisfied | Very Good | Excellent |
| --- | --- | --- | --- | --- |
| 1 | 2 | 3 | 4 | 5 |

4. A person with diabetes needs to exercise for 35-45 min, 4 times a week. Keeping this in mind how satisfied are you with the time you spend exercising?

| Very dissatisfied | Moderately dissatisfied | Neither satisfied nor dissatisfied | Very Good | Excellent |
| --- | --- | --- | --- | --- |
| 1 | 2 | 3 | 4 | 5 |

**General Health**

1. In general would you say your health is?

| Poor | Fair | Good | Very Good | Excellent |
| --- | --- | --- | --- | --- |
| 1 | 2 | 3 | 4 | 5 |

2. How well are you able to concentrate in everything like working , driving, reading etc?

| Not at all | A little | Moderate | Very Much | An Extreme Amount |
| --- | --- | --- | --- | --- |
| 1 | 2 | 3 | 4 | 5 |

3. How many times in the past three month have you had fatigue/ felt very tired?

| Poor | Fair | Good | Very Good | Excellent |
| --- | --- | --- | --- | --- |
| 1 | 2 | 3 | 4 | 5 |

**Symptom Botherness**

1. How many times in the past three months have you had thirsty/dry mouth?

| Always | Frequently | Often | Sometimes | Never |
| --- | --- | --- | --- | --- |
| 1 | 2 | 3 | 4 | 5 |

2. How many times in the past three months have you felt excessive hunger?

| Always | Frequently | Often | Sometimes | Never |
| --- | --- | --- | --- | --- |
| 1 | 2 | 3 | 4 | 5 |

3. How often in the last three months have you had frequent urination related to diabetes management?

| Always | Frequently | Often | Sometimes | Never |
| --- | --- | --- | --- | --- |
| 1 | 2 | 3 | 4 | 5 |

**Financial Worries**

1. What do you think about the cost involved in your management of diabetes?

| Very Expensive | Little expensive | Reasonable | Not at all expensive |
| --- | --- | --- | --- |
| 1 | 2 | 3 | 4 |

2. To what extent has your priority of expenditure shifted towards diabetes management?

| A lot | Highly | Little | Very little | Not at all |
| --- | --- | --- | --- | --- |
| 1 | 2 | 3 | 4 | 5 |

3. To what extent has your family budget got affected by the expenses related to the management of diabetes?

| A lot | Highly | Little | Very little | Not at all |
| --- | --- | --- | --- | --- |
| 1 | 2 | 3 | 4 | 5 |

4. To what extent has your diabetes limited your expenditure on the other aspects of life (Movies, outings, parties etc)?

| A lot | Highly | Little | Very little | Not at all |
| --- | --- | --- | --- | --- |
| 1 | 2 | 3 | 4 | 5 |

**Emotional and Mental health**

1. How satisfied are you with yourself?

| Very dissatisfied | Moderately dissatisfied | Neither satisfied not dissatisfied | Moderately satisfied | Very satisfied |
| --- | --- | --- | --- | --- |
| 1 | 2 | 3 | 4 | 5 |

2. How satisfied are you with your personal relationships (family, friends relatives, known tos)?

| Very dissatisfied | Moderately dissatisfied | Neither satisfied not dissatisfied | Moderately satisfied | Very satisfied |
| --- | --- | --- | --- | --- |
| 1 | 2 | 3 | 4 | 5 |

3. How satisfied are you with your emotional support you get from your family and friends?

| Very dissatisfied | Moderately dissatisfied | Neither satisfied not dissatisfied | Moderately satisfied | Very satisfied |
| --- | --- | --- | --- | --- |
| 1 | 2 | 3 | 4 | 5 |

4. How often are you discouraged by your health problems?

| Always | Frequently | Often | Sometimes | Never |
| --- | --- | --- | --- | --- |
| 1 | 2 | 3 | 4 | 5 |

5. All people want to fulfill certain roles and lead their lives in a purposeful manner, To what extent do you feel that you have been able to lead your life in the same way?

| Not at all | A little | Moderate | Very much | An extreme amount |
| --- | --- | --- | --- | --- |
| 1 | 2 | 3 | 4 | 5 |

**Diet Satisfaction**

1. How often do you feel because of your diabetes a restriction in choosing your food when eating out?

| Always | Frequently | Often | Sometimes | Never |
| --- | --- | --- | --- | --- |
| 1 | 2 | 3 | 4 | 5 |

2. As you have diabetes, how much choice do you feel you have in wating your meals or snacks away from home e.g. If you go in a party and there is a buffet where there are also a lot of fried snacks and desserts would you be able to make enough choice?

| No choice | Very little | Little | Enough | A lot |
| --- | --- | --- | --- | --- |
| 1 | 2 | 3 | 4 | 5 |

3. How often do you eat the food items that you shouldn’t, in other to hide the fact that you are having diabetes?

| Always | Frequently | Often | Sometimes | Never |
| --- | --- | --- | --- | --- |
| 1 | 2 | 3 | 4 | 5 |

**Physical Endurance** :

1. How often in last three months has your overall health problems limited the kind of vigorous activities you can do like lifting heavy bags/objects, running, skipping, jumping.

| Always | Frequently | Often | Sometimes | Never |
| --- | --- | --- | --- | --- |
| 1 | 2 | 3 | 4 | 5 |

2. How often in last three months has your overall health problems limited the kind of moderate

activites you can do like moving a table, carrying groceries or utensils.

| Always | Frequently | Often | Sometimes | Never |
| --- | --- | --- | --- | --- |
| 1 | 2 | 3 | 4 | 5 |

3. How often in last three months has your overall health problems limited you from walking uphill

or climbing 1-2 hours.

| Always | Frequently | Often | Sometimes | Never |
| --- | --- | --- | --- | --- |
| 1 | 2 | 3 | 4 | 5 |

4. How often in last three months has your overall health problems limited you from walking 1-2 km

at a stretch.

| Always | Frequently | Often | Sometimes | Never |
| --- | --- | --- | --- | --- |
| 1 | 2 | 3 | 4 | 5 |

5. How often in last three months has your overall health problems limited you from bending, squatting, or turning.

| Always | Frequent | Often | Sometimes | Never |
| --- | --- | --- | --- | --- |
| 1 | 2 | 3 | 4 | 5 |

6. How often in last three months has your overall health problems limited you from eating,

dressing, bathing, or using the toilet.

| Always | Frequently | Often | Sometimes | Never |
| --- | --- | --- | --- | --- |
| 1 | 2 | 3 | 4 | 5 |

**Role Limitation Due to Physical Health**

1. How often do you miss your work because of your diabetes?

| Always | Frequently | Often | Sometimes | Never |
| --- | --- | --- | --- | --- |
| 1 | 2 | 3 | 4 | 5 |

2. A person with the diabetes has the requirement of adhering to a schedule for eating and taking regular medication. How often does this affect your work?

| Always | Frequently | Often | Sometimes | Never |
| --- | --- | --- | --- | --- |
| 1 | 2 | 3 | 4 | 5 |

3. How often does diabetes affect your efficiency at work ?

| Always | Frequently | Often | Sometimes | Never |
| --- | --- | --- | --- | --- |
| 1 | 2 | 3 | 4 | 5 |

4. How often do you find diabetes limiting your social life?

| Always | Frequently | Often | Sometimes | Never |
| --- | --- | --- | --- | --- |
| 1 | 2 | 3 | 4 | 5 |

5. To what extent do you avoid travelling (business tour, holiday, general outings) because of your diabetes?

| Always | Frequently | Often | Sometimes | Never |
| --- | --- | --- | --- | --- |
| 1 | 2 | 3 | 4 | 5 |

6. Compared to others of your age are your social activites (visiting friends/partying)

limited because of your diabetes?

| Always | Frequently | Often | Sometimes | Never |
| --- | --- | --- | --- | --- |
| 1 | 2 | 3 | 4 | 5 |
